# Surveillance on secular trends of incidence and mortality for device–associated infection in the intensive care unit setting at a tertiary medical center in Taiwan, 2000–2008: A retrospective observational study

**DOI:** 10.1186/1471-2334-12-209

**Published:** 2012-09-10

**Authors:** Yin-Yin Chen, Liang-Yu Chen, Seng-Yi Lin, Pesus Chou, Shu-Yuan Liao, Fu-Der Wang

**Affiliations:** 1 Department of Infection Control, Taipei Veterans General Hospital, Taipei, Taiwan; 2 Department of Nursing, Taipei Veterans General Hospital, Taipei, Taiwan; 3 Center for Geriatrics and Gerontology, Taipei Veterans General Hospital, Taipei, Taiwan; 4 Division of Infectious Diseases, Department of Medicine, Taipei Veterans General Hospital, Taipei, Taiwan; 5 School of Nursing, National Yang–Ming University, Taipei, Taiwan; 6 School of Medicine, National Yang–Ming University, Taipei, Taiwan; 7 Institute of Public Health, and Community Medicine Research Center, National Yang–Ming University, Taipei, Taiwan; 8 Division of Infectious Diseases, Department of Medicine, St. Mary’s Hospital Luodong; St. Marys Medicine Nursing and Management College, Luodong, Taiwan

**Keywords:** Surveillance, Secular trend, Device–associated infection, Intensive care unit, Infection control

## Abstract

**Background:**

Device–associated infection (DAI) plays an important part in nosocomial infection. Active surveillance and infection control are needed to disclose the specific situation in each hospital and to cope with this problem effectively. We examined the rates of DAI by antimicrobial-resistant pathogens, and 30–day and in–hospital mortality in the intensive care unit (ICU).

**Methods:**

Prospective surveillance was conducted in a mixed medical and surgical ICU at a major teaching hospital from 2000 through 2008. Trend analysis was performed and logistic regression was used to assess prognostic factors of mortality.

**Results:**

The overall rate of DAIs was 3.03 episodes per 1000 device–days. The most common DAI type was catheter–associated urinary tract infection (3.76 per 1000 urinary catheter–days). There was a decrease in DAI rates in 2005 and rates of ventilator–associated pneumonia (VAP, 3.18 per 1000 ventilator–days) have remained low since then (*p* < 0.001). The crude rates of 30–day (33.6%) and in–hospital (52.3%) mortality, as well as infection by antibiotic-resistant VAP pathogens also decreased*.* The most common antimicrobial-resistant pathogens were methicillin–resistant *Staphylococcus aureus* (94.9%) and imipenem–resistant *Acinetobacter baumannii* (*p* < 0.001), which also increased at the most rapid rate*.* The rate of antimicrobial resistance among *Enterobacteriaceae* also increased significantly (*p* < 0.05). After controlling for potentially confounding factors, the DAI was an independent prognostic factor for both 30–day mortality (OR 2.51, 95% confidence interval [CI] 1.99–3.17, *p* = 0.001) and in–hospital mortality (OR 3.61, 95% CI 2.10–3.25, *p* < 0.001).

**Conclusions:**

The decrease in the rate of DAI and infection by resistant bacteria on the impact of severe acute respiratory syndrome can be attributed to active infection control and improved adherence after 2003.

## Background

Surveillance of nosocomial infections (NIs) has become an integral part of infection control and quality assurance in many countries. Gastmeier *et al.* reported that effective surveillance could reduce the NI rate on average about 20–30% [1,2]. Surveillance programs provide data on the microbes causing specific NIs and their resistance to antibiotics. Moreover, such programs can guide clinical practices and NI prevention efforts in different geographic regions and clinical settings.

The surveillance of device–associated infections (DAIs) in intensive care units (ICUs) has become more important owing to the more frequent employment of invasive advanced life support devices, especially after the introduction in 2004 of Surviving Sepsis Bundles [3,4]. Nevertheless, according to the three largest surveillance systems, the pooled mean rates of DAIs were: ventilator–associated pneumonia (VAP), 1.3–13.6 per 1000 ventilator–days; central line–associated bloodstream infection (CLABSI), 2.0–7.6 per 1000 catheter–days; and catheter–associated urinary tract infection (CAUTI), 2.0–6.3 per 1000 catheter–days [5-7]. In addition, DAIs have been associated with significant cost and mortality [3,5,6]. The crude mortality rates of ICU patients with DAI were 32.9–43.7% [5].

Moreover, as indicated by the message “Bad bugs, no drugs” released by the Infectious Disease Society of America in 2004, the emergence of antibiotic resistance threatens to exacerbate the problem of NIs in critically ill patients. Decreased susceptibility of both gram–positive and gram–negative microbes to antibiotics has been well described in several surveillance studies over the past decade, and increases in the rate of bloodstream infection caused by multi–drug resistant (MDR) gram–negative bacteria have been reported to be 16–fold [5,8-11]. In addition, both the morbidity and mortality rates have increased [12-14]. In this study, prospective surveillance was conducted to determine the DAI rate and prevalence of antibiotic-resistant isolates at an adult medical–surgical ICU (MS ICU). Our aim was to analyze the secular trend of incidence for different types of DAIs, determine the common pathogens involved, and determine the rates of antimicrobial resistance and overall 30–day and in–hospital mortality during the period 2000–2008.

## Methods

### Hospital and setting

This study was conducted in a 42–bed adult medical–surgical ICU with more than 1500 admissions (age 18 years or older) per year located in a 2900–bed major teaching hospital in the northern part of Taiwan. The hospital–wide infection surveillance and control program was established in 1982, with one infection control practitioner (ICP) for every 250 beds. All patients admitted to the ICU in the period 2000–2008 who developed infections more than 48 hours after admission (i.e., nosocomial infections) were eligible for the study. The protocol of this study was approved by the Institutional Review Board of our teaching hospital.

### Surveillance for nosocomial infection and data collection

This ICU-based surveillance was conducted according to the US Centers for Disease Control and Prevention (CDC) procedures. All patients in the unit were monitored for NIs that affected particular body sites. Infections at more than one site in the same patient were counted as separate infections. The antibiotic susceptibility of each pathogen involved was analyzed. The data were prospectively collected at least once a week in the ICU by trained ICPs according to standardized protocols and definitions of the US CDC National Healthcare Safety Network (NHSN; formerly the National Nosocomial Infection Surveillance system [NNIS]) [15]. All DAIs of the Outcome Surveillance Component were categorized using standard US CDC NHSN definitions that included laboratory and clinical criteria [16]. The involved patient demographic information, the dates and sites of infection, device–utilization (DU) ratio, pathogens, antimicrobial susceptibilities, invasive procedures, and overall 30–day mortality and in–hospital crude mortality were recorded. Reports of cases of DAI were also verified by an infectious disease specialist. Data were also collected for each exposed patient in the ICU from the prospective hospital database, including demographics and clinical characteristics.

### Definitions for nosocomial infection and device–associated infection

Pneumonia was defined when a patient had a new or progressive infiltrate, consolidation, cavitation, or pleural effusion on chest radiograph and had the following signs or symptoms: new onset of purulent sputum or change in character of sputum. A VAP was categorized as ventilator associated if the patient had been intubated and received ventilation for more than 48 hours prior to the development of pneumonia. To detect VAP microorganisms, tracheal aspirates obtained via endotracheal tube suction or tracheostomy tube suction methods were cultured.

Laboratory–confirmed bloodstream infection (BSI) was defined when a patient had a recognized pathogen cultured from one or more blood cultures *and* the microorganism cultured from blood was *not* related to an infection at another site. Common skin contaminants (e.g., coagulase–negative staphylococcus [CoNS]) required culture from two or more blood cultures drawn on separate occasions *or* at least one blood culture for a patient with intravascular devices and microorganisms of the tip culture identical to those of the blood culture.

A CLABSI was considered central catheter–associated if a catheter had been in place for more than 48 hours and a secondary site of infection was not present. To detect CLABSI micro–organisms, a central catheter were removed aseptically and a 5–cm segment from the most distal end of the tip of the catheter along with paired peripheral blood samples were cultured. Central catheter–tip colonization was defined as isolation of 15 colony–forming units from a central catheter tip by using the roll–plate semiquantitative Maki’s culture technique.

Symptomatic UTI was defined when a patient had one or more of the following signs or symptoms with no other recognized cause: fever (>38 °C), dysuria, urgency, frequency, or suprapubic tenderness and (1) the patient had a positive urine culture, that is, ≥10^5^ microorganisms per cm^3^, or urine with no more than two species of microorganisms, or (2) pyuria (urine specimen with ≥10 white blood cells /mm^3^*and* a positive urine culture of ≥10^3^ and <10^5^ CFU/ml with no more than 2 species of microorganisms. A CAUTI was a symptomatic UTI that occurred in a patient who had an indwelling urinary catheter in place within the 48 hour period before the onset of the UTI. To detect CAUTI organisms, a urine sample was aseptically aspirated from the sampling port of a urinary catheter and cultured quantitatively.

### Microbiological identification and antimicrobial susceptibility

Pathogens were isolated from blood cultures using the BACTEC® NR–660 system (Becton Dickinson Diagnostic Instrument Systems, Spark, MD, USA) between 2000 and 2001 and using the BacT/ALERT 3D system (bioMérieux, Inc., Marcy l’Étoile, France) between 2001 and 2008. Pathogens were isolated from other specimens using standard methods specified by the Clinical Laboratory Standard Institute (CLSI) 2008 [17]. Antibiotic susceptibilities were determined using disk diffusion tests and interpreted according to the criteria specified by the CLSI 2008.

### Statistical analysis

The NI rate was defined as the number of NIs per 1,000 patient–days. Patient days were calculated as the number of ICU days of the non–NI cohort or the number of ICU days after the onset of NI. Device–associated infection rates were calculated as the number of device–associated infections for a specified body site per 1,000 device days. The DU ratio was calculated as the number of device–days per number of patient–days. Secular trends of DU ratio, antimicrobial resistant rates, 30–day mortality and in–hospital mortality rates were analyzed by chi–square test for linear trend. The overall and site–specific DAI rates were analyzed by Poisson regression analysis. Logistic regression with a stepwise forward approach was used to assess prognostic factors of mortality, while controlling for potentially confounding variables (i.e., demographics, invasive devices, and laboratory data) [12]. Odds ratios (OR) and 95% confidence intervals (CI) were calculated. A *p*–value < 0.05 was defined as statistically significant. Statistical analysis was conducted using Epi info^TM^ version 3.5.3 released by US CDC. Graphs of secular trends, 30–day mortality and inhospital mortality rates were created using SigmaPlot version 10.0 (Systat Inc., San Jose, CA, USA).

## Results

During the study period, 126,315 patient–days and 275,067 device–days were evaluated, and 2,054 NIs and 833 DAIs occurred in 14,734 patients admitted to MS ICUs with a mean APACHE II score of 23.6 ± 7.2. The crude mortality rate was 14.4% during the study period. Those patients who were admitted to MS ICUs had a mean age of 69.5 ± 16.9 years, and male gender accounted for 71.8%. The length of ICU stay was 9.6 ± 7.5 days in average. Most patients were admitted due to major medical conditions (59%), such as neoplasms (22.2%), digestive system problems (20.7%), and respiratory system problems (17.8%). Patients with serum albumin <2.5 g/dL were 44.4% and blood creatinine >1.5 mg/dL were 67.1%. There were 13.2% patients undergoing hemodialysis during their ICU stay.

**Figure 1:**
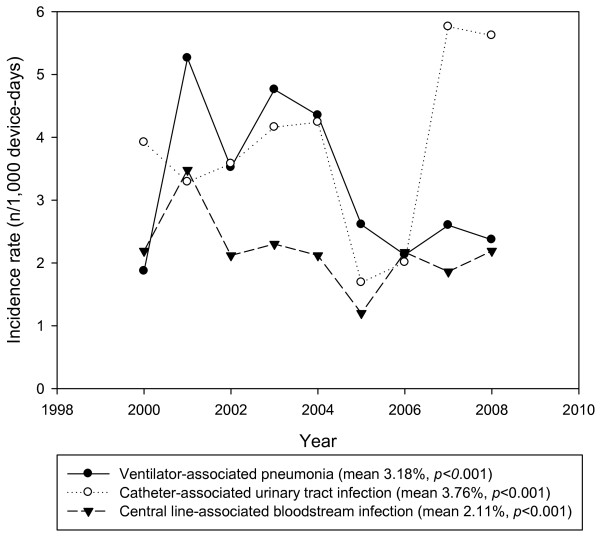
Trends of device-associated infections rates from 2000 to 2008.

The overall rates of NIs and DAIs were 16.26 episodes per 1000 patient–days and 3.03 episodes per 1000 device–days, respectively. The most common DAI type was CAUTI (mean 3.76 [352 episodes in 93,652 urinary catheter–days], range 1.69–5.76 per 1000 urinary catheter–days), followed by VAP (mean 3.18 [292 episodes in 91,911 ventilator–days], range 1.87–5.26 per 1000 ventilator–days), and CLABSI (mean 2.11 [189 episodes in 89,504 central line–days], range 1.20–3.48 per 1000 central line–days). DU ratios for mechanical ventilation (mean 0.73 [91,911 ventilator–days in 126,315 patient–days], *p* < 0.001) and central line catheterization (mean 0.71 [89,504 central line–days in 126,315 patient–days], *p* < 0.001) increased especially after 2005, but the incidence of VAP decreased until 2005 and then remained stable (*p* < 0.001) (Table 1 and Figure 1).

**Table 1:** Incidence and device–utilization ratio in device–associated infections from 2000 to 2008

**Device type**	**2000**	**2001**	**2002**	**2003**	**2004**	**2005**	**2006**	**2007**	**2008**	**Overall**	***p*value**
Patient days	13,915	14,144	14,144	14,021	14,274	14,206	13,753	14,049	13,809	126,315	
Ventilator–associated pneumonia, n	21	47	32	44	41	29	23	30	25	292	
Ventilator–days	11,211	8,941	9,101	9,246	9,420	11,121	10,778	11,545	10,548	91,911	
Rate per 1000 ventilator–days	1.87	5.26	3.52	4.76	4.35	2.61	2.13	2.60	2.37	3.18	<0.001
DU ratio	0.81	0.63	0.64	0.66	0.66	0.78	0.78	0.82	0.76	0.73	<0.001
Catheter–associated urinary tract infection, n	48	37	33	38	40	19	22	64	51	352	
Urinary catheter–days	12,231	11,233	9,208	9,140	9,426	11,268	10,965	11,104	9,077	93,652	
Rate per 1000 urinary catheter–days	3.92	3.29	3.58	4.16	4.24	1.69	2.01	5.76	5.62	3.76	<0.001
DU ratio	0.88	0.79	0.65	0.65	0.66	0.79	0.80	0.79	0.66	0.74	0.166
Central line–associated bloodstream infection, n	28	26	16	18	16	15	25	22	23	189	
Central line–days	12,773	7,480	7,531	7,819	7,546	12,494	11,502	11,833	10,526	89,504	
Rate per 1000 central line–days	2.19	3.48	2.12	2.30	2.12	1.20	2.17	1.86	2.19	2.11	<0.001
DU ratio	0.92	0.53	0.53	0.56	0.53	0.88	0.84	0.84	0.76	0.71	<0.001

Device–utilization (DU) ratio=number of device–days / number of patient–days.

A total of 1,290 pathogens were isolated from clinical specimens (Table 2). *Acinetobacter baumannii* (23%), *Pseudomonas aeruginosa* (18.2%), and *Staphylococcus aureus* (17.4%) were the three most common pathogens associated with VAP, while *S. aureu*s (20.4%), *A. baumannii* (14.9%), and *Candida albicans* (14.6%) accounted for the majority of CLABSIs. In contrast, non–albicans *Candida* (NAC) spp. (31%) rather than bacteria were the most common CAUTI pathogens, followed by *Enterococci* (10.1%) and *Escherichia coli* (9.9%).

**Table 2:** Pathogens isolated from device–associated infections during 2000–2008

**Isolated pathogens**	**Ventilator–associated pneumonia**	**Central line–associated bloodstream infection**	**Catheter–associated urinary tract infection**
	**(%)(n = 500)**	**(%)(n = 323)**	**(%)(n = 467)**
**Gram–positive cocci**	18.0	30.2	15.9
*Staphylococcus aureus*	17.4*	20.4*	2.6
coagulase–negative staphylococcus	0	4.6	1.5
*Enterococcus* sp.	0	3.4	10.5*
others	0	1.5	0.4
**Gram–positive bacilli**	0.6	0.3	0.9
**Gram–negative bacilli**	79.6	44.7	48.2
Enterobacteriaceae	14.6	11.8	25.3
*Escherichia Coli*	2.4	2.8	9.9*
*Klebsiella pneumoniae*	9.4	5.0	9.2
*Proteus Mirabilis*	0.2	0.3	2.6
*Serratia marcescens*	1.0	3.7	1.7
Enterobacter sp.	1.6	0	1.9
non fermentative Gram negative bacilli	62.2	27.3	19.9
*Pseudomonas aeruginosa*	18.2*	5.6	8.8
*Acinetobacter baumannii*	23.0*	14.9*	6.9
*Burkholderia cepacia*	4.8	2.2	1.3
*Stenotrophomonas maltophilia*	12.4	3.1	1.3
others	3.8	1.5	1.7
other Gram–negative bacilli	2.8	5.5	2.9
**Yeast**	2.4	25.1	35.9
*Candida Albicans*	0.2	14.6*	4.9
*non–albicans Candida spp.*	0.2	6.2	3.4
others	2.0	4.3	27.6*

*The first three common pathogens isolated in site–specific device–associated infection.

The rate of antibiotic resistance every year is presented in Table 3. The rate of infection by methicillin–resistant *S. aureus* (MRSA, mean 94.9% [243 cases in 256 patients with *S. aureus*]) remained relatively stable throughout the study period, and the rate of infection by resistant Gram–negative bacilli increased markedly from 2003. Particularly, the antibiotic-resistant proportion among *Enterobacteriaceae* infections increased significantly (*p* < 0.05), including ceftazidime–resistant *Klebsiella pneumoniae* (mean 58.7%, 64 cases in 109 patients with *K. pneumoniae*), ciprofloxacin–resistant *E. coli* (mean 47.4%, 37 cases in 78 patients with *E. coli*) and ceftazidime–resistant *E. coli* (mean 37.2%, 29 cases in 78 patients with *E. coli*).

**Table 3:** Antimicrobial resistant rates of common pathogens in adult medical–surgical intensive care unit during 2000–2008

**Isolated pathogen**	**Resistant rate (%)**										***p*value**
	**2000**	**2001**	**2002**	**2003**	**2004**	**2005**	**2006**	**2007**	**2008**	**Overall**	
*Staphylococcus aureus,* n	74	47	38	26	18	7	20	17	9	256	
Methicillin–R *S. aureus*	94.6	91.5	100	96.2	100	85.7	100	94.1	77.8	94.9	0.414
*Escherichia coli*, n	12	9	9	11	8	6	7	8	8	78	
Ceftriaxone–R *E. coli*	50	22.2	22.2	18.2	50	33.3	28.6	62.5	50	37.2	0.314
Ceftazidime–R *E. coli*	25	22.2	33.3	18.2	62.5	33.3	42.9	62.5	50	37.2	0.039*
Ciprofloxacin–R *E. coli*	–	22.2	33.3	54.5	75	83.3	71.4	87.5	37.5	47.4	0.046*
ESBL E. coli	–	–	–	–	–	–	14.3	50.0	50.0	39.1	0.176
*Klebsiella pneumoniae,* n	9	16	9	8	17	8	14	17	11	109	
Ceftriaxone–R *K. pneumoniae*	66.7	18.8	55.6	75	52.9	75	71.4	88.2	36.4	58.7	0.053
Ceftazidime–R *K. pneumoniae*	22.2	25.0	44.4	75	76.5	75	71.4	88.2	36.4	58.7	0.002*
Ciprofloxacin–R *K. pneumoniae*	–	43.8	33.3	37.5	70.6	50	78.6	58.8	27.3	48.6	0.507
ESBL *K. pneumoniae*	–	–	–	–	–	–	42.9	64.7	27.3	47.6	0.532
*Pseudomonas aeruginosa,* n	25	14	15	16	16	23	21	23	18	171	
Ceftazidime–R *P. aeruginosa*	24	14.3	13.3	12.5	6.3	26.1	28.6	17.4	16.7	18.7	0.846
Cefepime–R *P. aeruginosa*	–	–	–	6.3	6.3	0	0	0	11.1	2.3	0.715
Imipenem–R *P. aeruginosa*	8	14.3	6.7	0	12.5	22	0	0	16.7	8.8	0.812
*Acinetobacter baumannii,* n	22	32	28	33	21	19	23	21	26	225	
Imipenem–R *A. baumannii*	0	9.4	7.1	30.3	38.1	15.8	17.4	14.3	65.4	22.2	<0.001*

R: resistant, ESBL: Expanded Spectrum Beta-Lactamase producing strains.

*p < 0.05.

–: antibiotic susceptibilities in that year were not determined using disk diffusion tests.

**Figure 2:**
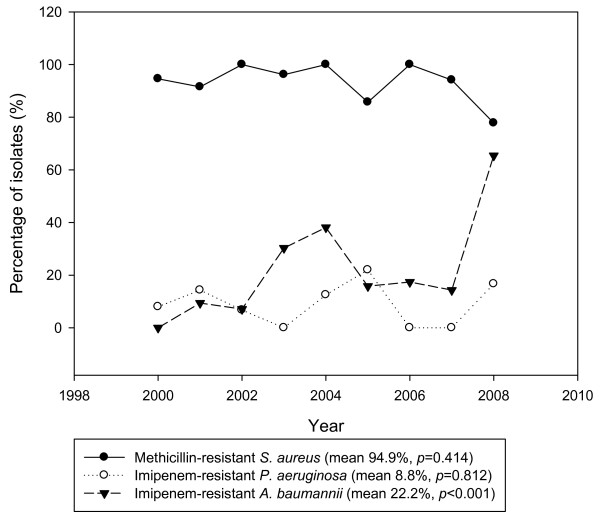
Trends of antimicrobial resistance rates among three common pathogens in ventilator-associated pneumonia.

Figure 2 shows the three most common VAP pathogens. The percentage of MRSA (mean 92.7%, 89 cases in 96 patients with *S. aureus*), imipenem–resistant *P. aeruginosa* (IRPA, mean 4.5% [5 cases in 110 patients with *P. aeruginosa*]), and imipenem–resistant *A. baumannii* (IRAB, mean 21.5% [29 cases in 135 patients with *A. baumannii*]) tended to decrease in 2005–2006. However, VAP–isolated IRAB in 2008 was increased. The percentage of isolates of MRSA (*p* = 0.678) and IRPA (*p* = 0.953), but not isolates of IRAB (*p* < 0.001), remained relatively constant.

**Figure 3:**
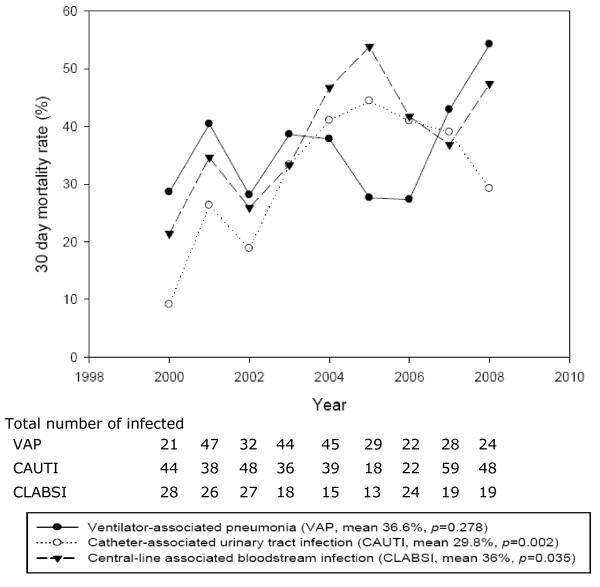
Thirty-day mortality in device-associated infections during 2000 to 2008.

**Figure 4:**
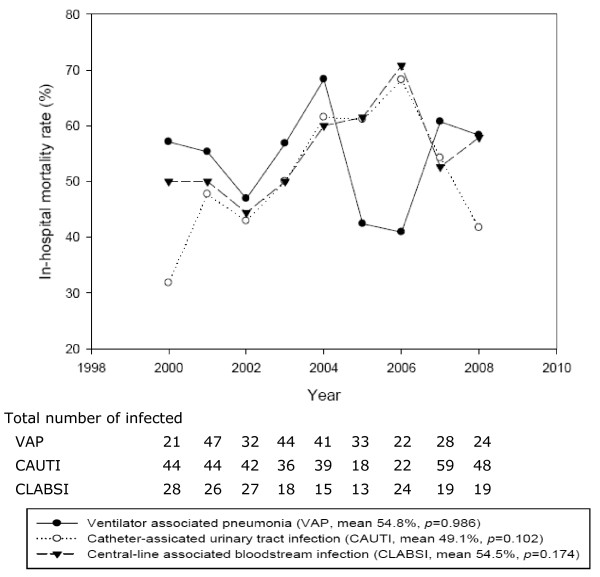
In-hospital mortality device-associated infections during 2000 to 2008.

The 30–day crude mortality rate and in–hospital crude mortality rate were 33.6% (280 died in 833 patients, range 17.2–40.4%) and 52.3% (436 died in 833 patients, range 43.0–64.2%), respectively. The 30–day *versus* in–hospital mortality rates for three site–specific DAIs were 36.6% (107 died in 292 patients) *versus* 54.8% (160 died in 292 patients) for VAP, 36.0% (68 died in 189 patients) *versus* 54.5% (103 died in 189 patients) for CLABSI, and 29.8% (105 died in 352 patients) *versus* 49.1% (173 died in 352 patients) for CAUTI. The overall trends of 30–day mortality rate showed significant variations (p < 0.05) in CAUTIs and CLABSIs, but in–hospital mortality rate for all DAIs were not significantly different (p > 0.05). Furthermore, 30–day and in–hospital mortality rates for VAP dropped markedly in 2005–2006, but the mortality rates increased after 2006 (Figures 3 and 4).

DAI was an independent factor for 30–day mortality (OR 2.51, 95% CI 1.99–3.17, *p* = 0.001) and in–hospital mortality (OR 2.61, 95% CI 2.10–3.25, *p* < 0.001) by multiple regression analysis. Other significant prognostic factors (*p* < 0.001) for mortality included APACHE II score, service, length of stay after the onset of infection, serum albumin, blood creatinine, neoplasms and hemodialysis (Table 4).

**Table 4:** Prognostic factors of mortality by logistic regression analyses

**Variables**	**Within 30-day mortality**			**In-hospital mortality**		
	**OR**	**95% CI**	***P*-value**	**OR**	**95% CI**	***P*-value**
Device-associated infection (yes/no)	2.51	1.99-3.17	<0.001	2.61	2.10-3.25	<0.001
APACHE II (every score)	1.05	1.04-1.06	<0.001	1.05	1.04-1.06	<0.001
Service (medical/surgical)	1.64	1.44-1.88	<0.001	1.68	1.40-1.96	<0.001
Length of stay after the onset of infection (every day)	1.04	1.03-1.05	<0.001	1.02	1.01-1.03	<0.001
Serum albumin (< 2.5 g/dL)	1.57	1.38-1.79	<0.001	1.57	1.37-1.78	<0.001
Blood creatinine ( >1.5 mg/dL)	1.68	1.44-1.96	<0.001	1.68	1.44-1.95	<0.001
Neoplasms (yes/no)	1.52	1.30-1.77	<0.001	1.44	1.24-1.68	<0.001
Hemodialysis whenever during ICU stay (yes/no)	1.38	1.15-1.77	0.001	1.38	1.16-1.65	<0.001

OR, Odds Ratio; CI, Confidence interval; APACHE, Acute Physiology and Chronic Health Evaluation.

## Discussion

The mean rates of NI and DAI in our adult MS ICU during the study period were much lower than those reported by the INICC as well as for 173 ICUs in developing countries [5], were similar to those reported by 1,545 hospitals in the US through the CDC NHSN [3,6], and were slightly higher than those indicated by 586 ICUs in the German Surveillance System (ICU-KISS) [7]. Reasons for these high DAI rates in the INICC report and developing countries may include resource limitations, lack of legal enforcement of the infection control program, and poor adherence to infection control guidelines [5]. The prospective hospital–wide surveillance and infection control program has been established for nearly 30 years in our hospital, which made a great effort to control infection by implementing infection control bundles and educational programs. The increase of device-related infections is not obvious after 2004, except for CAUTI. These strategies showed effectiveness in controlling DAI rates and suggest the necessity of infection control bundles implementation.

The common device–associated pathogens show geographic variation in distribution. *A. baumanniiS. aureus*, and *P. aeruginosa* were the three most common VAP pathogens in our study, the US CDC NHSN study, and the SENTRY antimicrobial surveillance study, although their percentages differed between studies [18,19]. The percentage of isolates of MRSA (*p* = 0.678) and IRPA (*p* = 0.953), but not isolates of IRAB (*p* < 0.001) remained relatively constant. However, any variation in these percentages would not be statistically significant and might rather be due to chance than to an actual variation.

In contrast, higher rates of *A. baumannii* and *C. albicans* isolation compensated for the relatively low rates of CoNS and *Enterococcus* spp. isolation in cases of CLABSI, while *C. albicans* was replaced by NAC spp in cases of CAUTI. Differences in clinical setting, institution, study period, target population, and specific infection type might account in part for differences between studies. CoNS was less frequently identified in CLABSI, because our criteria were slightly different from the US CDC definition for laboratory–confirmed BSI. The CDC defined skin contaminant BSI in 1998 and 2004 as 'the common skin contaminant (e.g., CoNS) is cultured from at least one blood culture from a patient with an intravascular line, and the physician institutes appropriate antimicrobial therapy'. However, CoNS BSIs in our study were enrolled if the patient had only one blood culture of CoNS that was positive but then microorganisms cultured from the tip of the intravascular device that were also CoNS. The percentage of CoNS isolates was expected to be at least that of *S. aureus* isolates reported in previous studies [15,16,18,20].

However, the frequency of *A. baumannii* and *Candida* spp. in specimens from patients with CLABSI was also reported to be increasing in other hospitals in Taiwan as well as several Asian countries such as Turkey and Thailand [20-24], although the frequency of NAC spp. represented by only one *Candida* spp. has also been rising in specimens from patients with CAUTI. Early and empirical usage of broad spectrum antibacterial agents in critically ill patients and preemptive administration of fluconazole are common factors contributing to the increase in frequency of isolation of these relatively resistant pathogens [22,23,25]. Use of indwelling catheters increases susceptibility to those multi–drug resistant pathogens and is associated with biofilm formation [26,27].

The high prevalence of MRSA is a common problem worldwide, and this situation was much more severe in our institute. Our data showed a lower incidence density of *S. aureus* but a higher proportion of MRSA. The percentage of MRSA infections was 74.4–84.1% in the INICC report [5], 54.4–65.2% in the US CDC NHSN report [18], and, in the Asia–Pacific region, it was 38.2% in the SENTRY Antimicrobial Surveillance Program report (2003–2004) [28] and 20–85% in the Tigecycline Evaluation and Surveillance Trial (TEST) report [29]. MRSA rates were decreasing in many European countries but not in USA [18,29]. The more severe illnesses of patients and more frequent use of broad–spectrum antibiotics might account in part for the high rate of MRSA isolation from patients in ICU at major teaching hospitals [29,30]. Another possible explanation is the clonal spread of resistance genes or resistant strains [31,32], but molecular analysis will be needed to prove this hypothesis.

According to the infection control policies in our ICU, when a patient was admitted to the ICU, a multi–drug resistant (MDR) checklist was used to inquire about MDR pathogens including MRSA infection or colonization. If MRSA had been isolated, then contact precautions were implemented. Furthermore, we have promoted hospital–wide hand–washing activity from 2006 to the present. The infection control team also carries on the non-warning investigation of hand-washing and of isolation precautions in each season, and gives feedback of the results to the unit. Infectious disease doctors assist in carrying on the infectious disease treatment and the antibiotic use in the ICU. MRSA infection rates have been reduced by year from 2006. Interestingly, the rates of antibiotic resistance for pathogens other than MRSA was lower at our hospital than those in previously published reports and lower than those for all NIs reported by the TEST and SENTRY antimicrobial surveillance programs [8,19,28,33].

However, despite the carbapenems being the most active antimicrobials against Acinetobacter species, the increasing development of significant carbapenem resistance among Acinetobacter species has been reported [3,5,34]. In the present study, the average percentage of *A. baumannii* isolates resistant to imipenem was 22.2%. The rate of ICU patients with IRAB DAI has been rapidly rising (from 6.1% to 34.3%). Among *Enterobacteriaceae*, Ciprofloxacin–R *E. coli* and Ceftazidime–R *K. pneumoniae* from 2003, and Ceftazidime–R *E. coli* from 2004, had significant increases. This finding revealed that the resistance of gram-negative bacteria has increased, the development of which should require closely monitored.

Aside from the fact that DAI is an important prognostic factor of mortality., several previous studies have shown that the mortality rate attributed to DAI is 1.65–2.75 times higher than that attributed to no infection [13,35,36]. Our study supports the findings of these published reports. In the present study, the multiple regression analysis indicated that patients with DAIs (compared to patients with no HAI) had significantly increased likelihood of mortality (*p* < 0.05). Moreover, the annual 30–day mortality rates of CAUTIs and CLABSIs had significant changes over the period 2000 through 2008. These results may be caused by chance, because this study period did not change substantially in terms of medical care, novelty medical technology, and patient disease severity.

In addition to the above–mentioned findings, we used a multiple regression analysis approach to adjust covariables, in addition to demographics, invasive devices, and laboratory investigations. We also identified severity of illness using APACHE II scores as a predictor of mortality, with the results indicating that the hazard of mortality is associated with increasing scores. Patients who died with DAI infection were usually already severely ill and their existing illness, rather than the DAI, was often the main cause of death. Thus, an important prognostic factor was the severity of their illness, which resulted in an increased likelihood of mortality [14,25]. We also found that patients with blood creatinine over 1.5 mg/dL were the highest risk group for dying. Excluding an endogenous effect, the reason may be that many patients in this group were receiving hemodialysis with CVCs inserted.

The rates of DAIs of all types decreased during the period 2005–2006, but this decrease was maintained only for VAP. Similarly, decreased NIs and DAIs were reported by other epidemiological studies [3,6,19,37]. Effective control of respiratory tract infection, antibiotics stewardship, implementation of traffic control practices, improved adherence to hand hygiene and contact precaution practices as a result of the severe acute respiratory syndrome (SARS) pandemic in 2003 as well as decreased rate of resistance of MRSA, IRPA, and IRAB in 2005 might account for this decrease [37-39].

Some limitations of the present study should be noted. The study was performed at a single medical center. However, the results could be provided to the hospital as a part of the teaching or research mission. This study was a retrospective nine-year survey which might have some potential biases. In the analysis of long–term changes in infection rates or mortality rates, we must consider whether changes in the population, advances in laboratory diagnostic techniques, changes in exposure to risk factors, microbial culture and other factors lead to increased or decreased rates. However, there did not occur any outbreaks of DAIs during the study period, except for the SARS outbreak.

## Conclusions

We have presented here the secular trend of DAIs at our institution in northern Taiwan, and the great achievement of our infection control and surveillance program, which was the maintenance of a low DAI incidence despite high device–utilization ratios. The incidence of DAIs decreased in 2005. The incidence of VAP remained low, and the rate of antimicrobial resistance of the three most common pathogens causing VAP decreased. Implementation of infection control and traffic control bundles improved adherence to hand hygiene practices and antibiotic stewardship, and the impact of the SARS pandemic on adherence to these practices might explain the decrease in DAI incidence and rate of antibiotic resistance in 2005. This study also demonstrated that DAI was an important independent prognostic factor of mortality.

## Competing interests

The authors declare that they have no competing interests.

## Authors’ contributions

YYC participated in the design, data collection and analysis, and drafted the manuscript. LYC participated in the analysis and drafted the manuscript. SYL and PC commented on drafts of the manuscript. SYL participated in the data collection. FDW conceived of the project, participated in the design and helped to draft the manuscript. All authors approved the final manuscript.

## Pre-publication history

The pre-publication history for this paper can be accessed here:

http://www.biomedcentral.com/1471-2334/12/209/prepub
